# A simple method for simultaneous RP-HPLC determination of indolic compounds related to bacterial biosynthesis of indole-3-acetic acid

**DOI:** 10.1007/s10482-012-9838-4

**Published:** 2012-10-31

**Authors:** Michał Szkop, Wiesław Bielawski

**Affiliations:** Department of Biochemistry, Faculty of Agriculture and Biology, Warsaw University of Life Sciences–SGGW, Nowoursynowska 159, 02-776 Warsaw, Poland

**Keywords:** HPLC, Indolic compounds, IAA biosynthesis

## Abstract

In this short technical report, we present a fast and simple procedure for sample preparation and a single-run Reversed Phase High Performance Liquid Chromatography (RP-HPLC) determination of seven indoles (indole-3-acetic acid, indole-3-acetamide, indole-3-acetonitrile, indole-3-ethanol, indole-3-lactic acid, tryptamine and tryptophan) in bacterial culture supernatants. The separation of the analytes, after a single centrifugal filtration clean-up step, was performed using a gradient elution on a symmetry C8 column followed by fluorimetric detection (λ_ex_ = 280/λ_em_ = 350 nm). The calibration curves were linear for all of the studied compounds over the concentration range of 0.0625–125 μg mL^−1^ (*r* ^*2*^ ≥ 0.998) and the limits of detection were below 0.015 μg mL^−1^. The applicability of the method was confirmed by analysis of *Pseudomonas* *putida* culture supernatants.

## Introduction

**Fig. 1:**
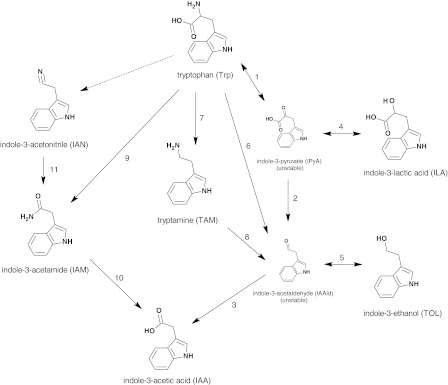
Simplified diagram of Trp-dependent pathways for IAA biosynthesis in bacteria (compiled from Carreno-Lopez et al. 2000; Spaepen et al. 2007). (1) Aromatic amino acid aminotransferase; (2) IPyA decarboxylase; (3) IAAld dehydrogenase; (4) ILA dehydrogenase; (5) TOL dehydrogenase; (6) Trp side-chain-oxidase; (7) Trp decarboxylase; (8) TAM oxidase; (9) Trp 2-mono-oxygenase; (10) IAM hydrolase; and (11) nitrilase

Indole-3-acetic acid (IAA) is the most abundant naturally occurring auxin, which is a class of phytohormones implicated in the regulation of plant growth and development. In addition to plants, diverse species of soil and plant-associated bacteria can also produce IAA. Through the identification of bacterial genes and enzyme activities and the detection of indolic intermediates in bacterial culture supernatants, the existence of five different IAA biosynthesis routes has been determined with tryptophan (Trp) as a precursor: the indole-3-pyruvate (IPyA), indole-3-acetamide (IAM), tryptamine (TAM), indole-3-acetonitrile (IAN) and the Trp side chain oxidase pathways (Fig. 1; described in Carreno-Lopez et al. 2000; Spaepen et al. 2007). Additionally, it was observed that IAA produced by bacteria via distinct pathways may differentially affect plant growth. While IAA biosynthesized by beneficial bacteria designated as PGPR via the IPyA intermediate stimulated root proliferation and growth, IAA produced by phytopathogenic bacteria via the IAM intermediate induced galls and tumors formation in plants (Lambrecht et al. 2000). These findings suggest that there is a link between the IAA biosynthetic pathway used by a given bacterial strain and the host plant phenotype. It is therefore important not only to verify the ability of a particular strain of bacteria to produce IAA but also to investigate the biosynthetic route of this compound through, together with above mentioned approaches, determination of other 3-substituted indoles in the bacterial culture supernatant.

Among the available methods to determine IAA and related compounds, spectrophotometric (Akbari et al. 2007; Karnwal 2009; Sahasrabudhe 2011), thin layer chromatography (TLC) (Hartmann et al. 1983; Robinson et al. 1998; Swain et al. 2007) and HPLC assays are used most frequently. Spectrophotometric methods, which are based on the reaction of indoles with the Salkowski reagent and compare the color development of the reaction mixture with an appropriate reference, are nonspecific and provide only quantitative determination of the total indoles content, rather than each of the analytes individually (Glickmann and Dessaux 1995). TLC methods, on the other hand, are used only for qualitative analysis. For both qualitative and quantitative determination of IAA and related 3-substituted indoles, several HPLC methods have been developed. However, these methods are complex and time- and reagent-consuming. First, sample preparation consists of several steps involving the repeated organic solvent extraction of an acidified bacterial culture supernatant (Garcia-Tabares et al. 1987; Sergeeva et al. 2007; Khakipour et al. 2008; Fedorov et al. 2010). It is worth noting that the extraction of TAM and indole-3-ethanol (TOL) from acidic solutions is difficult, and these compounds have been independently extracted from neutral or basic solutions (Crozier et al. 1988; Furukawa et al. 1996; Carreno-Lopez et al. 2000). Second, for HPLC separation of the studied group of indolic compounds, two independent runs with two different sets of eluents have been conducted (Manulis et al. 1994; Carreno-Lopez et al. 2000; Reineke et al. 2008).

Thus, the aim of the present study was to develop a fast, simple, and reliable method for the simultaneous preparation and a single-run RP-HPLC determination of indolic compounds related to bacterial IAA production. In our study, we included IAA (product), Trp (precursor) and all five known and stable intermediates of IAA biosynthesis: IAM, IAN, indole-3-lactic acid (ILA), TAM and TOL. ILA and TOL are products of the enzymatic reduction of IPyA and indole-3-acetaldehyde (IAAld), respectively. These latter two compounds are unstable and do not accumulate in bacterial cultures (Carreno-Lopez et al. 2000).

## Experimental

### Materials and reagents

Indole-3-acetic acid and *l*-tryptophan were purchased from Roth (Karlsruhe, Germany). Indole-3-acetamide, indole-3-acetonitrile, tryptamine, *DL*-indole-3-lactic acid, indole-3-ethanol and 3-kDa cut-off membrane centrifugal filters (Amicon Ultra 0.5 ml centrifugal filters Z677094) were obtained from Sigma-Aldrich Inc. (St. Louis, MO, USA). HPLC-grade acetonitrile and methanol were purchased from POCH SA (Gliwice, Poland). All aqueous solutions were prepared using ultra-pure Milli-Q water.

### Preparation of calibration curves and spike recovery analysis

The standard stock solutions (15 mg mL^−1^) of IAA, IAM, IAN, ILA, TAM and TOL were prepared in methanol. The standard Trp stock solution (15 mg mL^−1^) was prepared in water. A series (*n* = 13) of working solutions for each standard, ranging in concentration from 0.0625 to 125 μg mL^−1^, were prepared by appropriate dilution of the stock solution with methanol. All standard solutions were analyzed in triplicate. Calibration curves were constructed by performing a linear regression analysis of the peak area versus the analyte concentration. The limit of detection of each analyte was calculated from the chromatograms at a signal-to-noise ratio of 3.

To determine the recovery of the studied indolic compounds from bacterial broth during sample preparation, standards were spiked into sterile King B liquid medium (King et al. 1954) at three different concentrations. Bacterial broth was processed with the sample preparation procedure described below.

### Bacterial strain and culture conditions

*Pseudomonas putida* strain A, used in this study, was obtained from the collection of the Department of Biochemistry, SGGW, and was identified using the ribotyping method by Blirt S.A. DNA (Gdańsk, Poland). The 16S rRNA gene sequence of this strain is deposited in the DDBJ database under accession number AB667903. The bacteria were cultivated for 72 h in King B liquid medium with 0.5, 1.0, 2.0, 3.5 and 5.0 mM Trp supplementation. Bacterial cultures were then centrifuged, and the bacterial culture supernatants were processed with the sample preparation procedure described below.

### Sample preparation

Sample preparation consisted of a single centrifugal filtration step using 3-kDa cut-off membrane centrifugal filters. For this purpose, 0.5 mL of bacterial culture supernatants or spiked sterile bacterial broths were transferred to the sample chamber of a 0.5 mL centrifugal filter tube and centrifuged at 14,000×*g* (relative centrifugal force) at 4 °C for 30 min. The filtrates were directly analyzed by HPLC.

### Instrumentation and chromatographic conditions

The HPLC system was composed of a binary pump (Model 1525, Waters Corporation, Milford, MA, USA), a fluorimetric detector (Model 474, Waters), an autosampler (Model 717plus, Waters) and a personal computer with Breeze data acquisition and integration software (Waters). Chromatographic separations were performed at ambient temperature on a C8 column (Symmetry 4.6 × 150 mm, 5 μm, Waters) fitted with a C8 guard column (Symmetry 3.9 × 20 mm, 5 μm, Waters) using gradient elution. Eluent A consisted of 2.5 : 97.5 % (v/v) acetic acid : H_2_O, pH 3.8 (the pH was adjusted by addition of 1 mol L^−1^ KOH) and eluent B consisted of 80 : 20 % (v/v) acetonitrile : H_2_O. The mobile phase started with eluent A : eluent B at 80 : 20 %, changing to 50 : 50 %, 0 : 100 % and 80 : 20 % in 25, 31 and 33 min, respectively. The total run time was 36 min. The flow rate of the mobile phase was 1 mL min^−1^, the injection volumes were 20 μL, and the fluorimetric detector was set to excitation and emission wavelengths of 280 and 350 nm, respectively.

## Results and discussion

**Fig. 2:**
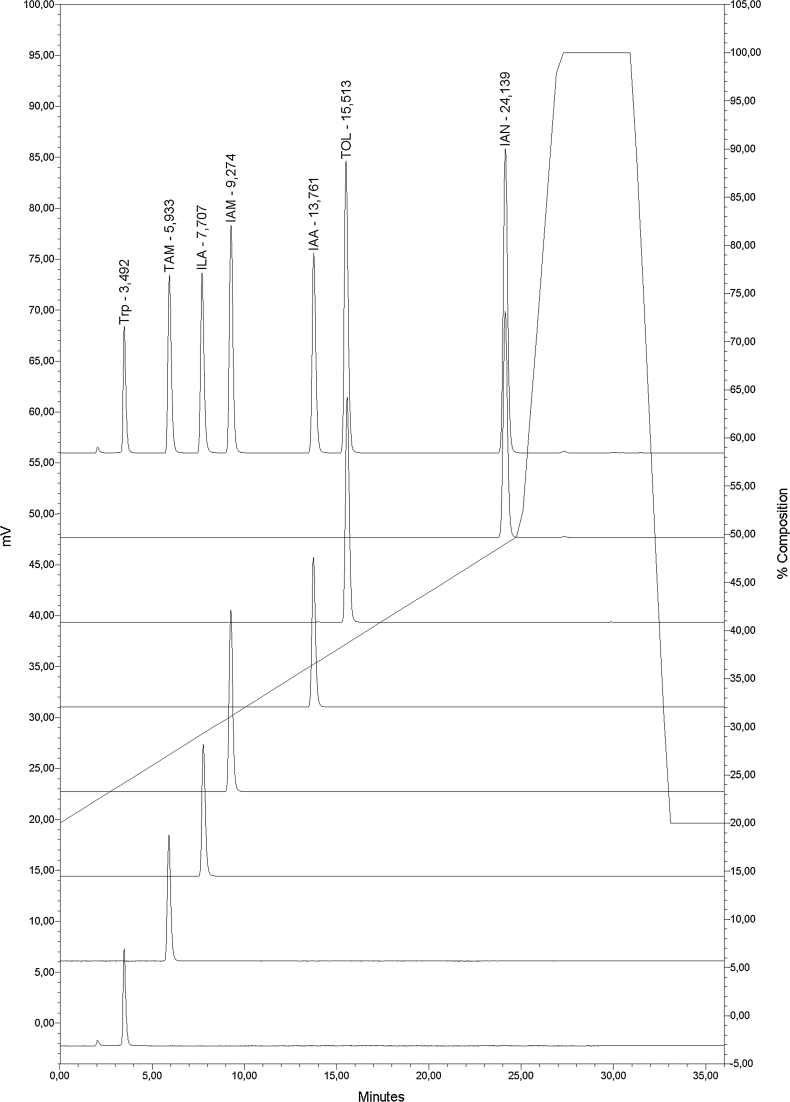
Representative chromatograms from the separation of indolic standards. Chromatogram on the *top* shows the separation of a mixture of all indolic standards (each at a concentration of 125 μg mL^−1^). Chromatograms from *top to bottom* show separation of each of the indolic standards (100 μg mL^−1^) separately. *Solid line* represents the gradient profile

**Fig. 3:**
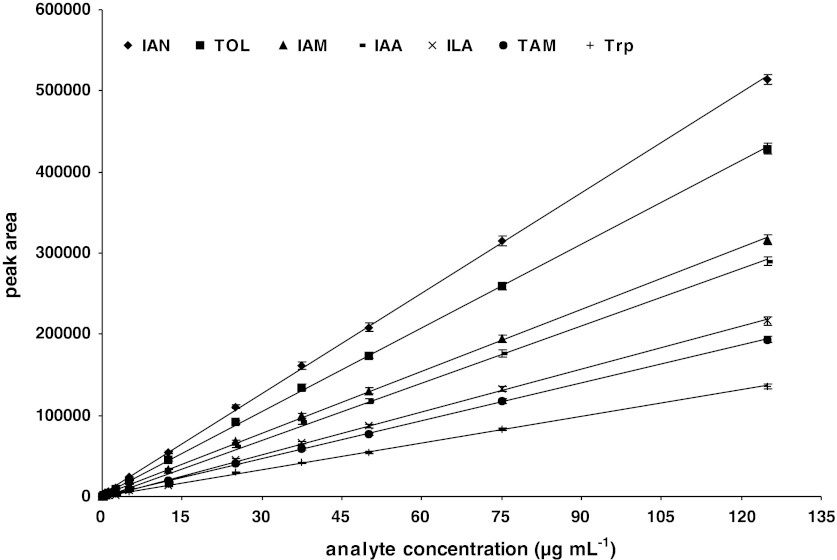
Calibration curves (analyte concentration vs peak area) for studied indolic compounds. Each data point represents the mean ± SD of three determinations

**Fig. 4:**
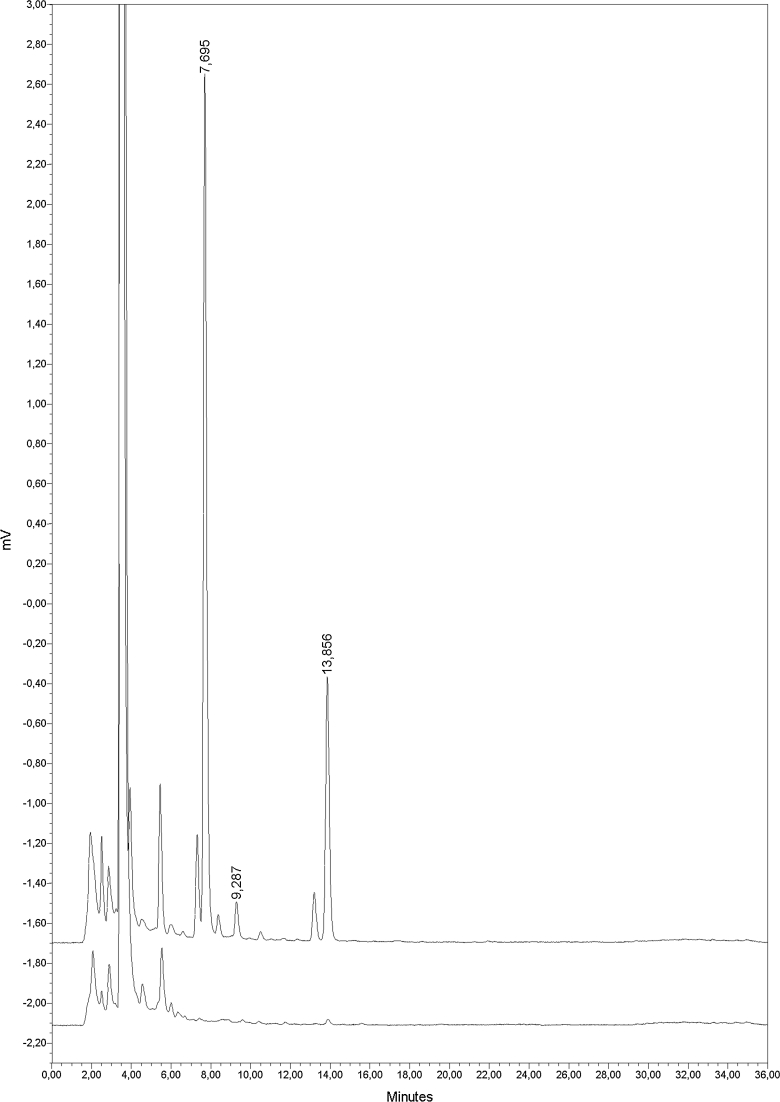
Representative chromatograms obtained during the analysis of indolic compounds in *P*. *putida* strain A culture supernatants. Chromatogram on the *top* shows the separation of *P*. *putida* strain A culture supernatant after 72 h of growth in King B medium supplemented with 3.5 mM Trp. Chromatogram on the *bottom* shows the separation of a sterile King B medium supplemented with 3.5 mM Trp (control)

Considering that the studied group of indoles possess acidic (IAA, ILA), amphoteric (Trp), basic (TAM) or essentially neutral (IAN, IAM, TOL) characters and that the pH of the mobile phase is an important factor influencing retention time and peak shape of ionizable compounds (Espinosa et al. 2002; Chandrul and Srivastava 2010), we tested mobile phases with different pHs, in the range of 2.5 to 7.5, with gradient or isocratic elution to determine the optimal chromatography conditions. The best conditions for separation were obtained using 2.5 : 97.5 % (v/v) acetic acid : H_2_O, pH 3.8 and 80 : 20 % (v/v) acetonitrile : H_2_O with gradient elution. Under these conditions, peaks were sharp, well resolved, and symmetrical for all analytes. The retention times were approximately 3.5, 5.9, 7.7, 9.3, 13.8, 15.5 and 24.1 min for Trp, TAM, ILA, IAM, IAA, TOL and IAN, respectively (Fig. 2). The linearity of the dependence of the detector response on the analyte concentration was evaluated by triplicate analysis of thirteen standard solutions containing 0.0625–125 μg mL^−1^ of each indolic compound (Fig. 3). All the resulting calibration curves were characterized by a high coefficient of determination (*r* ^*2*^ ≥ 0.998), indicating the broad linear working range of the method. The limits of detection for all studied analytes were below 0.015 μg mL^−1^ (Table 1). These results, together with the fact that the concentrations of indolic compounds accumulating in bacterial cultures typically reaches from a few to several dozens of μg mL^−1^ (Ahmad et al. 2005; Khakipour et al. 2008; Chaiharn and Lumyong 2011), indicate that no additional enrichment of the biological sample is required. Therefore, instead of time- and reagent-consuming organic solvent extraction, we utilized a single centrifugal filtration step for sample preparation. This simple step eliminates high molecular weight (> 3 kDa) contaminants that damage the column, gives excellent recoveries at all levels of analyte loading for all studied indolic compounds (Table 2) and provides adequate sample purity with low levels of interference (see chromatograms in Fig. 4).

**Table 1:** Calibration curves parameters and the limits of detection of studied indolic compounds

Compound	Retention time (min)	The linear regression equation	Coefficient of determination (*r* ^*2*^)	Limit of detection (μg mL^−1^)
Trp	3.5 ± 0.04	y = 1090.1x + 469.0	0.9997	0.0141
TAM	5.9 ± 0.04	y = 1547.4x + 495.1	0.9998	0.0099
ILA	7.7 ± 0.06	y = 1751.9x – 1044.8	0.9984	0.0096
IAM	9.3 ± 0.07	y = 2548.4x + 1442.3	0.9996	0.0079
IAA	13.8 ± 0.09	y = 2346.1x − 1116.3	0.9980	0.0089
TOL	15.5 ± 0.10	y = 3437.4x + 1618.7	0.9997	0.0062
IAN	24.1 ± 0.10	y = 4132.5x + 2080.0	0.9997	0.0043

**Table 2:** Recoveries of the indolic compounds from the spiked King B liquid medium (*n* = 4)

Compound	Amount added (μg mL^−1^)	Amount found (μg mL^−1^)	Recovery (%)
Trp	0.50	0.49	98.83 ± 2.1
	10.00	10.47	104.67 ± 1.9
	125.00	121.81	97.45 ± 1.6
TAM	0.50	0.47	94.24 ± 3.2
	10.00	9.94	99.44 ± 2.0
	125.00	122.98	98.38 ± 3.5
ILA	0.50	0.48	96.08 ± 4.5
	10.00	9.97	99.74 ± 2.1
	125.00	125.32	100.26 ± 2.4
IAM	0.50	0.48	96.70 ± 4.6
	10.00	10.24	102.44 ± 2.3
	125.00	124.71	99.77 ± 1.9
IAA	0.50	0.50	100.30 ± 4.1
	10.00	9.98	99.84 ± 2.8
	125.00	126.75	101.4 ± 2.7
TOL	0.50	0.47	94.09 ± 4.2
	10.00	9.99	99.87 ± 3.1
	125.00	123.28	98.62 ± 2.6
IAN	0.50	0.46	92.43 ± 4.0
	10.00	9.48	94.77 ± 2.3
	125.00	119.41	95.53 ± 2.9

**Fig. 5:**
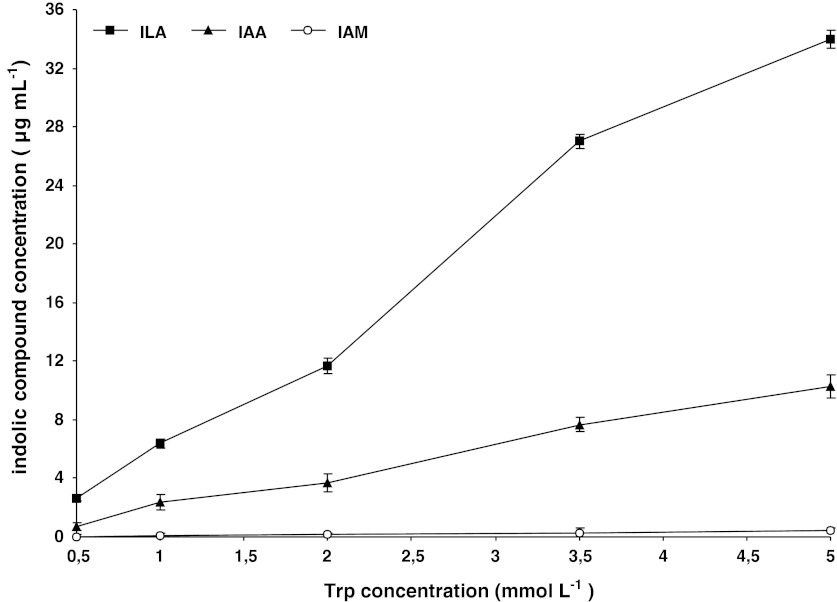
Effect of Trp concentration on ILA, IAA and IAM biosynthesis by *P*. *putida* strain A. Each data point represents the mean ± SD of three determinations

Finally, to verify the reliability of the established method, we analyzed culture supernatants of *P*. *putida* after 72 h of growth in King B medium supplemented with different concentration of Trp. In the tested samples, we identified 3 compounds that had retention times corresponding to those of ILA, IAA and IAM (Fig. 4). Quantitative analysis revealed that ILA was the most abundant compound (2.6–34.0 μg mL^−1^), followed by IAA (0.7–10.3 μg mL^−1^), with IAM present in very small amounts (0–0.4 μg mL^−1^). The level of biosynthesis of these compounds was positively correlated with Trp concentration (Fig. 5). These results are consistent with other reports on the effect of exogenously supplied Trp on the level of IAA (or other indoles) biosynthesis by bacteria (Carreno-Lopez et al. 2000; Sergeeva et al. 2007; Spaepen et al. 2007), which shows the consistency of the method. Moreover, detection of small amounts of IAM suggest that there is direct conversion of this compound to IAA, and that the studied *P*. *putida* strain operates via IAM pathway. Accumulation of high amounts of ILA (together with no detection of TOL) indicates, on the other hand, that Trp is efficiently metabolized via transamination, but resulting IPyA (ILA) is not further converted to IAA. These observation agrees with our previous findings concerning studied strain of bacteria, from which we isolated two aromatic amino acid aminotransferase isozymes with high activities toward Trp (Szkop et al. 2012).

## Summary

The method described here, compared to those applied previously, significantly simplifies both the sample preparation and HPLC analysis of IAA and related indolic compounds in bacterial culture supernatants. First, a simple one-step sample preparation procedure, taking advantage of the fluorescent properties of indoles, provides adequate sample purity, selectivity and sensitivity. Second, the developed and optimized chromatographic conditions allow for determination of a investigated range of indolic compounds in a single chromatographic run using one set of eluents. Therefore, the presented method should be especially useful for routine assays.
